# MicroRNA-33b knock-in mice for an intron of sterol regulatory element-binding factor 1 (*Srebf1*) exhibit reduced HDL-C *in vivo*

**DOI:** 10.1038/srep05312

**Published:** 2014-06-16

**Authors:** Takahiro Horie, Tomohiro Nishino, Osamu Baba, Yasuhide Kuwabara, Tetsushi Nakao, Masataka Nishiga, Shunsuke Usami, Masayasu Izuhara, Fumiko Nakazeki, Yuya Ide, Satoshi Koyama, Naoya Sowa, Naoya Yahagi, Hitoshi Shimano, Tomoyuki Nakamura, Koji Hasegawa, Noriaki Kume, Masayuki Yokode, Toru Kita, Takeshi Kimura, Koh Ono

**Affiliations:** 1 Department of Cardiovascular Medicine, Graduate School of Medicine, Kyoto University, Kyoto 606-8507, Japan; 2 Department of Clinical Innovative Medicine, Institute for Advancement of Clinical and Translational Science, Graduate School of Medicine, Kyoto University, Kyoto 606-8507, Japan; 3 Department of Internal Medicine (Endocrinology and Metabolism), Graduate School of Comprehensive Human Sciences, Nutrigenomics Research Group, Faculty of Medicine, and International Institute for Integrative Sleep Medicine (IIIS), World Premir International Research Center Initiative (WPI), University of Tsukuba, 1-1-1 Tennodai, Tsukuba, Ibaraki 305-8575, Japan; 4 Department of Pharmacology, Kansai Medical University, Moriguchi, Osaka 570-8506, Japan; 5 Division of Translational Research, National Hospital Organization, Kyoto Medical Center, Kyoto 612-8555, Japan; 6 Division of Clinical Pharmacy, Faculty of Pharmaceutical Sciences, Kobe Gakuin University, Kobe 650-8586, Japan; 7 Department of Cardiovascular Medicine, Kobe City Medical Center General Hospital, Kobe 650-0046, Japan; 8 These authors contributed equally to this work.

## Abstract

MicroRNAs (miRs) are small non-protein-coding RNAs that bind to specific mRNAs and inhibit translation or promote mRNA degradation. Recent reports, including ours, indicated that miR-33a located within the intron of sterol regulatory element-binding protein (SREBP) 2 controls cholesterol homeostasis and can be a possible therapeutic target for treating atherosclerosis. Primates, but not rodents, express miR-33b from an intron of *SREBF1*. Therefore, humanized mice, in which a miR-33b transgene is inserted within a *Srebf1* intron, are required to address its function *in vivo*. We successfully established miR-33b knock-in (KI) mice and found that protein levels of known miR-33a target genes, such as ABCA1, ABCG1, and SREBP-1, were reduced compared with those in wild-type mice. As a consequence, macrophages from the miR-33b KI mice had a reduced cholesterol efflux capacity via apoA-I and HDL-C. Moreover, HDL-C levels were reduced by almost 35% even in miR-33b KI hetero mice compared with the control mice. These results indicate that miR-33b may account for lower HDL-C levels in humans than those in mice and that miR-33b is possibly utilized for a feedback mechanism to regulate its host gene *SREBF1*. Our mice will also aid in elucidating the roles of miR-33a/b in different genetic disease models.

Sterol regulatory element-binding proteins (SREBPs) comprise a subclass of basic helix-loop-helix leucine zipper transcription factors conserved from yeasts to humans and regulate the expression of genes required for maintaining cellular lipid homeostasis^1^. Mammals possess two SREBP genes, SREBP-1 and SREBP-2 (known as *SREBF1* and *SREBF2*, respectively) that express three major SREBP proteins. Two SREBP-1 isoforms, SREBP-1a and SREBP-1c, primarily regulate fatty acid metabolism, and SREBP-2 is the main regulator of cholesterol metabolism, although there is some functional overlap among the three SREBP isoforms^2^,^3^,^4^.

MicroRNAs (miRs) are small non-protein-coding RNAs that bind to specific mRNAs and inhibit translation or promote mRNA degradation^5^. Recent advances in the understanding of miR biology revealed that the genetic loci encoding for the transcription factors SREBP-1 and SREBP-2 also encode for the miRs miR-33b and miR-33a, respectively. Recent reports, including ours, indicated that miR-33a controls ABCA1 expression and reduces HDL-C levels^6^,^7^,^8^ and that miR-33a deficiency ameliorates atherosclerosis in mice^9^,^10^,^11^. However, in rodents, a part of miR-33b is lacking from a *Srebf1* intron (Supplementary Fig. 1a), and it is impossible to determine the precise coordinate mechanisms of miR-33a and miR-33b; the expression of these miR-33s is expected to depend on their corresponding host genes. Of note, SREBP-1 and SREBP-2 are differentially regulated by hormones, dietary challenges, or statin treatment, and the amounts and functions of miR-33a and miR-33b would be greatly affected under these conditions.

miR-33a and miR-33b are identical in their seed sequences, and thus have been predicted to repress the same set of genes with similar specificities. Antisense oligonucleotides against miR-33a are believed to simultaneously target miR-33a and miR-33b. However, there remains a 2-nucleotide mismatch after the seed sequence between miR-33a and miR-33b (Supplementary Fig. 1a), and whether this difference results in differential targeting remains to be established. Moreover, some of the previously established miR-33a target genes were not dysregulated in our miR-33a-deficient mice. Therefore, humanized mice, in which a miR-33b transgene is inserted within a *Srebf1* intron, are required to address its function *in vivo*.

We successfully established miR-33b knock-in (KI) mice for the same intron as in humans. The protein levels of known miR-33a target genes, such as ABCA1, ABCG1, and SREBP-1, were reduced under basal conditions. An LXR agonist, which induces *Srebf1* expression, enhanced miR-33b production. *In vitro* experiments indicated that macrophages from the miR-33b KI mice had a reduced cholesterol efflux capacity via apoA-I and HDL-C. Moreover, HDL-C levels were reduced by almost 35% even in miR-33b KI hetero mice compared with the control mice.

The feasibility of genetic manipulation is one of the many advantages of using mice as a model organism. However, the lack of miR-33b in mouse *Srebf1* has raised an important concern regarding the direct translation of data from rodent models to human physiology and metabolic disorders. Our mice will aid in answering these questions and will be useful for assessing the risks and benefits of long-term alterations in miR-33s in different disease models. These mice might also be useful for screening of the drugs that alter the levels of miR-33a and miR-33b.

## Results

### miR-33b is co-expressed with *SREBF1* in the human cell line HepG2

**Figure 1:**
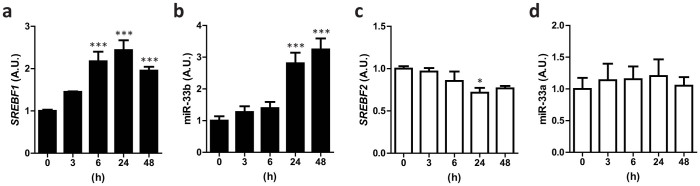
miR-33b is co-expressed with *SREBF1* in HepG2 cells. HepG2 cells were treated with T0901317 (10 μM) for the indicated time. The relative expressions of *SREBF1* (a), miR-33b (b), *SREBF2* (c), and miR-33a (d) are shown (n = 6–9). Values are mean ± s.e.m. *p < 0.05, ***p < 0.001 compared with 0 h.

It is assumed that a miR located within an intron of a gene is expressed along with its host gene and exerts its specific function^12^. Because miR-33b is located in a *SREBF1* intron in humans (Supplementary Fig. S1a), we stimulated human cell line HepG2 with the LXR agonist T0901317 and determined miR-33b and miR-33a expression along with the expression of the host genes *SREBF1* and *SREBF2*. As shown in Fig. 1a and b, miR-33b expression seemed to tag along behind *SREBF1* expression. In contrast, miR-33a and *SREBF2* expression was not affected by LXR stimulation (Fig. 1c and d).

### Generation of miR-33b KI mice

**Figure 2:**
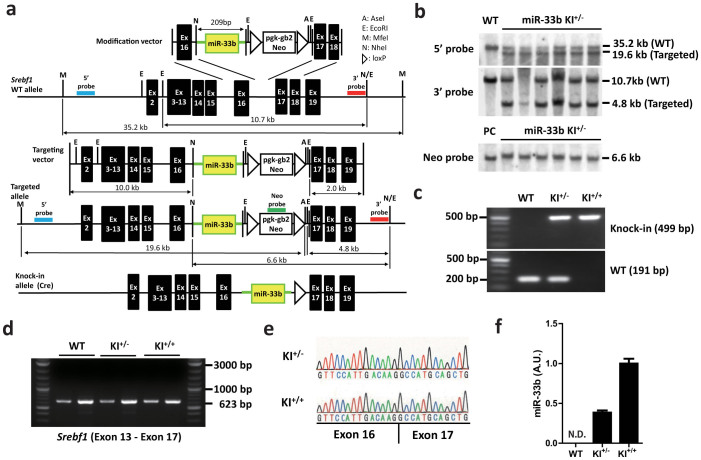
Generation of miR-33b knock-in (KI) mice. (a). Strategy used to generate miR-33b KI mice. (b). Southern blotting of mouse tail genomic DNA. Representative images are shown. (c). PCR analysis of mouse tail genomic DNA. Representative images are shown. (d). RT-PCR analysis of *Srebf1* expression in the livers of 8-week-old mice. Sense primer was designed for exon 13, and antisense primer was designed for exon17. Note that there was no other band except for that of the correct size. Representative images are shown. (e). Sequencing alignment at the joint between exons 16 and 17 of *Srebf1* in the indicated mice. (f). Relative expression of miR-33b in the livers of 8-week-old mice (n = 6). N.D., not determined. Values are mean ± s.e.m.

Because miR-33b is located in *SREBF1* intron 16 in humans and there are high homologies in exons 16 and 17 between human and mouse (82.6% of nucleotides and 79.7% of amino acids, Supplementary Fig. S1b), we introduced the human miR-33b sequence into intron 16 of mouse *Srebf1*. We isolated and amplified the region that encoded for the complete pre-miR sequence of human miR-33b and adjacent sequence, which enabled the introduction of miR-33b into intron 16 of mouse *Srebf1* (Fig. 2a). Supplementary Figure S2a and Figure 2b show the results of Southern blotting analysis of genomic DNA from ES cells and tail genomic DNA from F1 mice that were successfully targeted by a KI vector, respectively. PCR analysis indicated the specific patterns for wild-type (WT), KI^+/−^, and KI^+/+^ mice (Fig. 2c). This miR-33b KI strategy did not alter *Srebf1* intron 16 splicing, as confirmed by PCR (Fig. 2d) and sequencing (Fig. 2e). The expression levels of miR-33b in miR-33b KI^+/−^ mice were almost half of those in miR-33b KI^+/+^ mice (Fig. 2f). We also measured the levels of miR-33b, miR-33a, *Srebf1*, and *Srebf2* in WT and KI mice in both the liver and the peritoneal macrophages (Supplementary Figure S2b–d and S3a–d). *Srebf1* levels were similar among these mice (Supplementary Figure S2c and S3c). Although there was no difference in miR-33a levels in macrophages (Supplementary Figure S3b), miR-33a levels were increased in proportion of the expression levels of miR-33b in the liver (Supplementary Figure S2b). The miR-33b KI^+/+^ mice were born with the expected Mendelian ratios, were viable, fertile, and did not exhibit any obvious abnormalities in size, shape, or structure up to 8 weeks of age. Relative tissue expression pattern of miR-33b was similar to that of *Srebf1* (Supplementary Fig. S2e and S2f).

### miR-33b is upregulated after inducing *Srebf1* expression

**Figure 3:**
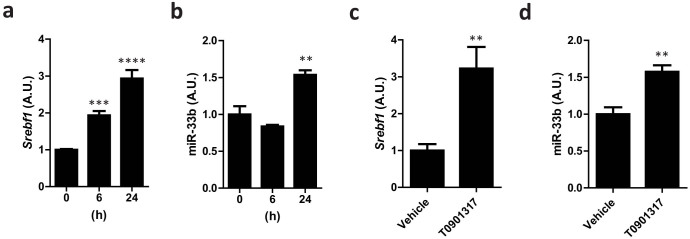
miR-33b is co-expressed with *Srebf1* in miR-33b KI mice. (a). Relative *Srebf1* expression levels in primary hepatocytes from miR-33b KI^+/+^ mice treated with T0901317 (10 μM) for the indicated time. Values are mean ± s.e.m (n = 6). **p < 0.01 ***p < 0.001 by one-way analysis of variance. (b). Relative miR-33b expression levels in primary hepatocytes from miR-33b KI^+/+^ mice treated with T0901317 (10 μM) for the indicated time. Values are mean ± s.e.m (n = 6). **p < 0.01 by one-way analysis of variance. (c). Relative *Srebf1* expression levels in the livers of 8-week-old male miR-33b KI^+/+^ mice treated with T0901317 (25 mg/kg) for 3 days. Values are means ± s.e.m (n = 6). **p < 0.01 compared with the vehicle. (d). Relative miR-33b expression levels in the livers of 8-week-old male miR-33b KI^+/+^ mice treated with T0901317 (25 mg/kg) for 3 days. Values are mean ± s.e.m (n = 6). *p < 0.05 compared with the vehicle.

We next sought to confirm whether miR-33b expression was affected by endogenous changes in *Srebf1* expression by the LXR agonist T0901317^13^. When primary hepatocytes from the miR-33b KI^+/+^ mice were stimulated with T0901317, *Srebf1* and miR-33b mRNA levels were significantly increased in parallel, although this increase was faster for *Serbf1* than for miR-33b (Fig. 3a and b). To check this effect *in vivo*, T0901317 was suspended in 0.5% carboxymethylcellulose and administrated to 8-week-old male miR-33b KI^+/+^ mice at a dose of 25 mg/kg for 3 days. The average liver weight of the T0901317-treated mice was 1.5-fold greater than that of the control mice (Supplementary Fig. S4a). *Srebf1* and miR-33b expression levels in the liver were also significantly increased in parallel (Fig. 3c and d). The average liver weight and *Srebf1* expression level in the liver of T0901317-treated WT mice were shown in Supplementary Figure S4b and S4c. These results indicate that miR-33b was co-expressed with *Srebf1* in the livers of the T0901317-treated miR-33b KI mice.

### miR-33b KI results in alterations in miR-33a target proteins ABCA1 and SREBP-1

**Figure 4:**
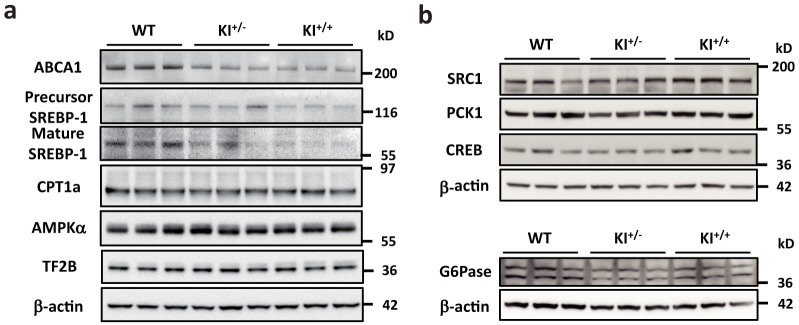
miR-33b regulates ABCA1 and SREBP-1. (a). Western blotting analysis for ABCA1, SREBP-1, CPT1a, and AMPKα protein levels in the livers of WT, KI^+/−^, and KI^+/+^ mice. Representative images are shown. TF2B and β-actin were used as loading controls. (b). Western blotting analysis for SRC1, PCK1, CREB and G6Pase protein levels in the livers of WT, KI^+/−^, and KI^+/+^ mice. Representative images are shown. β-actin were used as loading controls.

We determined ABCA1, SREBP-1, CPT1a, and AMPKα protein levels in the liver (Fig. 4a and Supplementary Figure S5). As shown in Fig. 4a, Supplementary Figure S5a, and S5b, ABCA1 and SREBP-1 protein levels were lower in the livers of the miR-33b KI mice. However, the protein levels of some of the previously defined miR-33a target genes, such as CPT1a and AMPKα remained unchanged. We also analyzed protein expressions of glucose metabolic genes (Fig. 4b and Supplementary Figure S5c–f). However, no significant change in protein level was observed in PCK1, G6PC, and CREB in the liver of miR-33b KI mice compared with that of control mice. SRC1 was up-regulated in miR-33bKI mice and it was opposite to the results of previous report^14^.

### miR-33b KI reduces cholesterol efflux in macrophages

**Figure 5:**
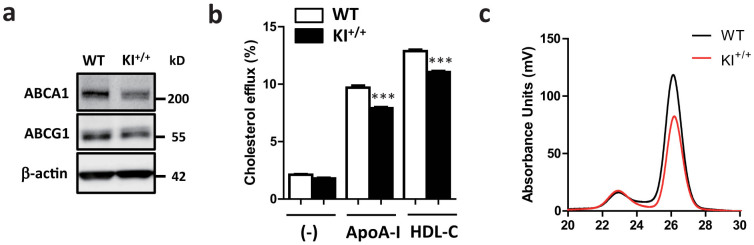
miR-33b reduces cellular cholesterol efflux and serum HDL-C levels. (a). Western blotting for ABCA1 and ABCG1 proteins in peritoneal macrophages from WT and KI^+/+^ mice. Representative images are shown. β-actin was used as the loading control. (b). Cholesterol efflux to apoA-I and HDL-C in peritoneal macrophages from WT and KI^+/+^ mice (n = 6 each). Values are mean ± s.e.m. ***p < 0.001 (c). Mean plots of HPLC analysis for serum cholesterol in WT and KI^+/+^ mice (n = 4 and 5, respectively).

To investigate a physiological role of miR-33b in mice, we first compared the functions of peritoneal macrophages from the WT and miR-33b KI^+/+^ mice. ABCA1 and ABCG1 protein levels were lower in macrophages from the miR-33b KI^+/+^ mice than from the WT mice (Fig. 5a and Supplementary Figure S3e and S3f), which was compatible with the findings for our miR-33a-deficient mice. We determined apoA-I- and HDL-C-mediated cholesterol efflux from peritoneal macrophages and found that macrophages from the miR-33b KI^+/+^ mice had lower apoA-I- and HDL-C-mediated cholesterol efflux than those from the WT mice (Fig. 5b).

### A single miR-33b copy reduces serum HDL levels

Hepatic ABCA1 overexpression increases HDL-C levels^15^, and liver-specific deletion of ABCA1 results in a substantial decrease in plasma HDL-C levels (approximately 80%) in chow-fed mice^16^. Moreover, we previously reported that the miR-33a^−/−^ mice had 22%–39% higher serum HDL-C levels than the WT mice^8^. Thus, we determined the serum HDL-C levels of the WT, miR-33b KI^+/−^, and miR-33b KI^+/+^ mice at the age of 8 weeks.

Serum HDL-C levels were significantly decreased in the miR-33b KI^+/−^ and miR-33b KI^+/+^ mice compared with the WT mice (Table). We also classified and quantified serum lipoproteins using high-performance liquid chromatography (HPLC). Mean plots of the HPLC elution profile of serum from male mice are shown in Fig. 5c, and the lipid profiles are summarized in Supplementary Table S1. These results show that only one copy of miR-33b was sufficient to substantially reduce HDL-C and total cholesterol to the same levels as those in the miR-33b KI^+/+^ mice. Moreover, the decreased HDL levels mainly comprised very large-, large-, and medium-sized HDLs (mature HDLs) (Fig. 5c and Supplementary Table S1).

## Discussion

In the present study, we successfully established humanized mice, in which a miR-33b transgene was inserted within the same intron as that in human *SREBF1*. The LXR agonist T0901317, which is a well-established *Srebf1* expression inducer, enhanced miR-33b production. The protein levels of known miR-33a/b target genes, such as ABCA1, ABCG1, and SREBP-1, were reduced under basal conditions. *In vitro* experiments indicated that macrophages from the miR-33b KI^+/+^ mice had a reduced cholesterol efflux capacity via apoA-I and HDL-C. Finally, HDL-C levels were reduced by almost 35% even in the miR-33b KI^+/−^ mice compared with the WT mice without any changes in triglyceride (TG) levels.

In contrast to humans and other mammals, rodents lack miR-33b and only have miR-33a in *Srebf2*. This needs to be kept in mind when attempting to directly translate to humans the previous results that miR-33a inhibition could prevent atherosclerosis in mouse models because of two reasons. First, *SREBF1* and *SREBF2* are differentially regulated by hormones, dietary challenges, and lipid-lowering agents, including statins^17^. This indicates that both isoforms of miR-33 participate in regulating the primary risk factors of metabolic syndrome, which accelerate atherosclerosis. Second, miR-33b differs from miR-33a by 2-nucleotides and may have a different target profile, including stronger effects on targets in the SREBP-1-dependent regulation of fatty acid/TG homeostasis and insulin signaling. We found increased miR-33b expression after treatment with LXR agonist in our mice, which indicated that miR-33b was co-expressed with its *Srebf1* host gene and enabled us to study the impact of *Srebf1*-derived miR-33b on cholesterol/lipid homeostasis.

We have not yet succeeded in identifying miR-33b-specific target genes. Even previously reported miR-33b target genes were not reduced in the liver of miR-33b KI mice compared with that of control mice. One of the reasons of such result may be that the previous study was conducted in human cell line and potential binding sites of miR-33b are not conserved at least in PCK1 3′UTR of mice. It is also possible that some compensated mechanisms may have occurred in miR-33b KI mice. However, the protein levels of miR-33a target genes, such as ABCA1, ABCG1, and SREBP-1, were reduced^18^. Moreover, the protein levels of previously defined miR-33a target genes, which were not dysregulated in miR-33a KO mice, including CPT1a and AMPKα, remained unchanged^19^,^20^. Thus, it may be necessary to assess those conditions when *Srebf1* expression is strongly affected to establish the importance of the functions of miR-33b. In any event, the numbers of miR-33b transcripts were greater than those of miR-33a transcripts, and this underscores the importance of miR-33b^21^. Although there were no differences in the levels of miR-33a in macrophages, it is interesting that the levels of miR-33a were increased in proportion to the expression levels of miR-33b in the liver. Because *Srebf1* level is higher in the liver than that in macrophages^22^, it is possible that miR-33b and miR-33a compete for the same target gene binding sites in the liver, and that the degradation of miR-33a is inhibited by miR-33b expression. In addition, there may be other unknown mechanisms.

miRs are known to target long non-coding RNAs whose functions are largely unknown, and interactions between miRs are also possible^23^. Thus, miR-33b-specific functions should be determined in future experiments.

Rayner *et al.* recently showed that inhibiting miR-33a and miR-33b in healthy male non-human primates increased circulating HDL-C levels^21^. More recently, Rottiers *et al.* reported that miR-33a and miR-33b acted in a redundant manner and that inhibiting both isoforms by an 8-mer LNA-modified anti-miR enhanced HDL-C levels^24^. Our data demonstrated that miR-33b indeed functions to control HDL-C levels, which highlights the importance of targeting both miR-33 family members simultaneously. It is noteworthy that only one copy of miR-33b (miR-33b KI^+/−^ mice) significantly reduced HDL-C levels to the same levels as those in the miR-33b KI^+/+^ mice. This explains one of the reasons why human HDL-C levels are lower than those of mice and indicates that it is important to considerably reduce miR-33b levels if pharmacological targeting of miR-33s is used to increase HDL-C levels. In this context, the current LNA-modified anti-miR technique is quite potent for reducing the levels of both miR-33 isoforms and may be useful for anti-atherosclerosis therapy.

In addition to the effects on HDL-C, a study by Rayner *et al.* showed that miR-33 antagonism reduced very low-density lipoprotein-associated TGs in their cohort of normal male African green monkeys^21^. However, Rottiers *et al.* did not find any significant changes in TG levels when using miR-33a/b-targeting LNA-anti-miR treatment^24^. In our present miR-33b KI study and in previous miR-33a KO experiments^8^, we did not observe any changes in TG levels, indicating that modulation of miR-33s is unlikely to have a strong effect on TG levels, although species differences and different dietary conditions need to be considered.

In contrast, we found a significant inhibitory effect of miR-33b on SREBP-1. A feedback system of SREBP-2 by cholesterol levels is well known, which maintains appropriate levels of cellular cholesterol. However, a similar mechanism has not been established for SREBP-1. Chronic activation of SREBP-1c in cases of overnutrition can lead to serious obesity-related problems. miR-33b may be utilized for a feedback mechanism to regulate its host gene *SREBF1* because insulin induces hepatic SREBP-1c expression and promotes lipogenesis and hepatic TG synthesis (Supplementary Fig. S6).

In the present study, we demonstrated the effect of miR-33b on HDL-C levels *in vivo*. We assume that inhibiting both miR-33a and miR-33b will have a significant effect on HDL-C levels in clinical settings. However, it is known that one miR can have hundreds of target genes and unexpected side effects may occur due to long-term therapeutic modulation of miR-33 to cure metabolic diseases. Careful observations of miR-33b KI and miR-33a-deficient mice and intercrossing of these mice will enable us to detect miR-33a- and miR-33b-specific target genes and to elucidate the overall functions of miR-33a and miR-33b *in vivo*. Moreover, our mice will aid in analyzing the roles of miR-33a/b in different genetic disease models and in screening drug candidates that can modulate miR-33a and miR-33b levels and activities.

## Methods

### Materials

The following antibodies were used: anti-ABCA1 (NB400-105) and anti-ABCG1 (NB400-132) (Novus Biologicals, Littleton, CO, USA); anti-AMPKα (#2532) and anti-CREB (#9197) (Cell Signaling Technology, Beverly, MA, USA); anti-CPT1a (ab128568) and anti-PCK1 (ab70358) (Abcam, Cambridge, UK); anti-β-actin (AC-15; A5441, Sigma-Aldrich, St. Louis, MO, USA); anti-SREBP-1 (sc-13551, sc-8984), anti-SRC1 (sc-8995), anti-G6Pase (sc-27198), and anti-TF2B (sc-225) (Santa Cruz, Biotechnology, California, USA). Anti-mouse, anti-rabbit and anti-goat IgG HRP-linked antibodies were purchased from GE Healthcare (Amersham, UK). Human apoA-I was purchased from Sigma-Aldrich. Human acetylated LDL (acLDL) and human HDL-C were purchased from Biomedical Technologies, Inc. (Stoughton, MA, USA). [1, 2-^3^H (N)]-Cholesterol was purchased from Perkin Elmer (Boston, MA, USA). T0901317 was purchased from Cayman Chemical (Ann Arbor, MI, USA).

### Generation of miR-33b KI mice

A targeting vector was constructed by modifying bacterial artificial chromosome RP24-310C22 (Invitrogen) using a defective prophage λ-Red recombination system^25^,^26^. As a selection marker, a neomycin resistance cassette flanked by loxP sites (loxP-PGK-gb2-neo-loxP cassette; Gene Bridges, Germany) was inserted at the adjacent site of the human pre-miR-33b site. The targeting vector was electroporated into C57BL/6 mouse ES cells (DS Pharma Biomedical) using a Nucleofector system (Lonza). Positive clones were selected by incubating cells with 200 μg/ml geneticin (Invitrogen) for 5 days, and homologous recombination was confirmed by Southern blotting. Successfully recombined ES cells were injected into blastocysts from ICR strain mice supplied by Unitech Inc. (Japan), and chimeric mice were bred with C57BL/6 mice to generate F1 mice. F1 mice genotypes were confirmed by Southern blotting. The neomycin resistance cassette was removed from the mouse germ line by breeding heterozygous mice with *Ayu-1 Cre* KI mice, which expressed Cre recombinase in multiple tissues, including the germ line^27^. Descendant miR-33b knock-in heterozygous mice without the *Ayu-1 Cre* allele were crossed with each other to generate the miR-33b KI^+/+^ mice. All experiments were performed with male C57BL/6 background mice and wild-type littermates were used as a control. All of the experimental protocols were approved by the Ethics Committee for Animal Experiments of Kyoto University and the methods were performed in *accordance* with the guidelines approved by the ethics committee. Primers used for genotyping were as follows: WT/KI sense, ATGGATTTACCTCAGTTTTAACGAC; WT antisense, CATCACTGAAGCACTGCATCTGC; KI antisense, AAGTGGATCCAGAATTCGTGA; *Cre* sense, GCTGCCACGACdCAAGTGACAGCAATG; and *Cre* antisense, GTAGTTATTCGGATCATCAGCTACAC.

### Southern blotting

Southern blotting was performed using DIG High Prime DNA Labeling and Detection Starter Kit II (Roche) according to the manufacturer's protocol. Genomic DNA samples were purified and digested with MfeI and AseI for a 5′ probe, EcoRI for a 3′ probe, and NheI for a Neo probe. Primer sequences used to amplify these probes were as follows:

5′ probe sense, CACGGTTGTGAGAAGTCAGTATTC; 5′ probe antisense, CTTTGCAAGCTCCTTGAGAATAAG; 3′ probe sense, AGTAAAATTCTCCTCAATGAACGTG; 3′ probe antisense, CAGTAGGTGACATTGTGATTGATCT; Neo probe sense, GAACAAGATGGATTGCACGCAGGTTCTCCG; and Neo probe antisense, GTAGCCAACGCTATGTCCTGATAG.

### Determination of splicing between exons 16 and 17 in *Srebf1*

We amplified the fragment between *Srebf1* exons 13 and 17 using cDNA from the livers of the indicated mice by PCR, and these products were then electrophoresed. Extension time was sufficient to expand the fragment when the correct splicing did not occur. There was no other band except for that of the correct size. Sequencing was performed using a primer for exon 16 and an ABI 3130 genetic analyzer. Primer sequences used were as folows:

Exon 13 sense, CCTAGAGCGAGCGTTGAACT; Exon 17 antisense, CTACCTGGACTGAAGCTGGTG; and Exon 16 sequence primer, AGGGCAGCTCTGTACTCCTTC.

### Cell culture

HepG2 cells were cultured in Dulbecco's modified Eagle's medium (DMEM; Nacalai Tesque, Japan) supplemented with 10% fetal bovine serum (FBS). Mouse primary hepatocytes were obtained from miR-33b KI^+/+^ mice using a two-step collagenase perfusion method^28^. In brief, hepatocyte suspensions were obtained by passing a collagenase type II (Gibco BRL, Life Technologies Inc., Rockville, MD, USA)-digested liver sample through a 70-μm cell strainer, followed by centrifugation to isolate mature hepatocytes. Hepatocytes were then resuspended in DMEM supplemented with 10% FBS and seeded on collagen type I-coated dishes (Iwaki Asahi Glass Co. Ltd., Japan) at a density of 7 × 10^4^ cells/ml. After incubation for 24 h, the cells were used for experiments.

### Cholesterol efflux from macrophages

Cellular cholesterol efflux via apoA-I was determined as described previously^29^. In brief, thioglycollate-elicited mouse peritoneal macrophages were plated in 24-well microplates at a density of 5 × 10^6^ cells/ml. Cells were cultured for 24 h in RPMI 1640 containing ^3^H-labeled acLDL (1.0 μCi/ml of ^3^H-cholesterol and 25 μg/ml of acLDL). On the next day, the cells were washed 3 times with RPMI 1640 and incubated for 6 h in RPMI 1640 with or without apoA-I (10 μg/ml) or HDL (100 μg/ml). Cholesterol efflux was expressed as the percentage of radioactivity released from cells in medium relative to the total radioactivity in cells plus medium.

### Western blotting

Western blotting was performed using standard procedures as described previously^30^. A lysis buffer was supplemented with a complete mini protease inhibitor (Roche), ALLN (25 μg/ml), 0.5 mM NaF, and 10 μM Na_3_VO_4_ just prior to use. Protein concentrations were determined using a bicinchoninic acid protein assay kit (Bio-Rad). All samples (20 μg of protein) were suspended in lysis buffer, fractionated using NuPAGE 4%–12% Bis-Tris (Invitrogen) gels, and transferred to a Protran nitrocellulose transfer membrane (Whatman). The membrane was blocked using 1× phosphate-buffered saline (PBS) containing 5% non-fat milk for 1 h and incubated with a primary antibody [anti-ABCA1 (1:1000), anti-ABCG1 (1:1000), anti-AMPKα (1:1000), anti-SREBP-1 (1:250), anti-TF2B (1:1000), anti-β-actin (1:3000), anti-CREB (1:1000), anti-PCK1 (1:1000), anti-SRC1 (1:200), anti-G6Pase (1:200) or anti-CPT1a (1:1000)] overnight at 4°C. After washing with PBS–0.05% Tween 20 (0.05% T-PBS), the membrane was incubated with a secondary antibody (anti-rabbit, anti-mouse and anti-goat IgG HRP-linked; 1:2000) for 1 h at 4°C. The membrane was then washed with 0.05% T-PBS and detected with an ECL Western Blotting Detection Reagent (GE Healthcare) using an LAS-1000 system (Fuji Film).

### RNA extraction and quantitative RT-PCR (qRT-PCR)

Total RNA was isolated and purified using TriPure Isolation Reagent (Roche). cDNA was synthesized from 1 μg of total RNA using the Transcriptor First Strand cDNA Synthesis Kit (Roche) according to the manufacturer's instructions. For qRT-PCR, specific genes were amplified in 40 cycles using SYBR**™** Green PCR Master Mix (Applied Biosystems). Expression was normalized to that of the housekeeping gene β-actin. Gene-specific primers are as follows;

*SREBF1* sense, AACAGTCCCACTGGTCGTAGAT; *SREBF1* antisense, TGTTGCAGAAAGCGAATGTAGT; *SREBF2* sense, AGGAGAACATGGTGCTGA; *SREBF2* antisense, TAAAGGAGAGGCACAGGA; *ACTB* sense, AGGCACTCTTCCAGCCTTCC; *ACTB* antisense, GCACTGTGTTGGCGTACAGG; *Srebf1* sense, TAGAGCATATCCCCCAGGTG; *Srebf1* antisense, GGTACGGGCCACAAGAAGTA; *Srebf2* sense, GTGGAGCAGTCTCAACGTCA; *Srebf2* antisense, TGGTAGGTCTCACCCAGGAG; *Actb* sense, GATCTGGCACCACACCTTCT; and *Actb* antisense, GGGGTGTTGAAGGTCTCAAA.

### Quantitative PCR for miRs

Total RNA was isolated using the TriPure Isolation Reagent (Roche). miR-33a and miR-33b were measured using TaqMan MicroRNA assay protocols (Applied Biosystems). Products were analyzed using a thermal cycler (ABI Prism® 7900HT sequence detection system). miRs expression of samples were normalized by U6 snRNA expression.

### Serum biochemical analysis

After mice were fasted for 4–6 h, blood was obtained from the inferior vena cava of an anesthetized mouse, and serum was separated by centrifugation at 4°C and stored at −80°C. Employing standard methods, biochemical measurements were made using a Hitachi 7180 Auto Analyzer (Nagahama Life Science Laboratory, Nagahama, Japan). Lipoproteins were analyzed by HPLC at Skylight Biotech (Akita, Japan), according to the procedures described previously^31^.

### Statistical analysis

Results are given as mean ± s.e.m. Statistical comparisons were made using unpaired two-tailed Student's *t*-tests or one-way analysis of variance with the Bonferroni post hoc test, as appropriate. A p value of <0.05 was considered significant.

## Author Contributions

T.H., T.N. and K.O. designed the project; T.H., T.N., O.B., Y.K., T.N., M.N., S.U., M.I., F.N., Y.I., S.K. and N.S. performed experiments; T.H., T.N., N.Y., H.S., T.N., K.H., N.K., M.Y., T.K., T.K. and K.O. analyzed and interpreted data; and T.H., T.N. and K.O. prepared the manuscript.

## Supplementary Material

Supplementary Information Supplementary Information

## Figures and Tables

**Table 1:** Serum profiling of WT, KI^+/−^, and KI^+/+^ mice

	WT (n = 4)	KI^+/−^ (n = 4)		KI^+/+^ (n = 4)	
TP (g/dL)	4.375 ± 0.1109	4.275 ± 0.04787		4.350 ± 0.05000	
ALB (g/dL)	2.950 ± 0.1190	2.825 ± 0.1109		2.900 ± 0.04082	
BUN (mg/dL)	21.75 ± 0.6801	20.58 ± 1.248		21.58 ± 1.680	
CRE (mg/dL)	0.1125 ± 0.002500	0.0925 ± 0.004787	*	0.0975 ± 0.006292	
Na (mEq/L)	152.5 ± 0.6455	153.5 ± 0.2887		153.8 ± 0.4787	
K (mEq/L)	3.350 ± 0.05000	3.325 ± 0.0750		3.350 ± 0.1041	
Cl (mEq/L)	110.5 ± 0.6455	110.8 ± 0.2500		111.0 ± 0.5774	
Ca (mg/dL)	8.500 ± 0.1871	8.325 ± 0.1109		8.350 ± 0.08660	
IP (mg/dL)	7.775 ± 0.4589	7.225 ± 0.2955		7.400 ± 0.4637	
T-BIL (mg/dL)	0.0875 ± 0.004787	0.0925 ± 0.008539		0.0825 ± 0.01109	
AST (IU/L)	39.25 ± 1.702	33.50 ± 1.658		39.25 ± 1.702	
ALT (IU/L)	26.50 ± 3.663	21.00 ± 2.415		22.75 ± 1.702	
ALP (IU/L)	505.5 ± 48.55	398.5 ± 40.01		480.0 ± 29.31	
LDH (IU/L)	278.3 ± 77.21	243.5 ± 55.30		255.0 ± 55.26	
AMY (IU/L)	2295 ± 68.22	2224 ± 62.39		2363 ± 97.02	
γ-GTP (IU/L)	3>	3>		3>	
T-CHO (mg/dL)	98.50 ± 5.694	66.25 ± 2.287	**	62.00 ± 1.225	***
TG (mg/dL)	34.75 ± 2.780	32.25 ± 3.065		35.25 ± 4.328	
NEFA (μEq/L)	471.0 ± 47.36	474.8 ± 71.81		459.5 ± 55.01	
LDL-C (mg/dL)	6.750 ± 0.6292	6.750 ± 0.6292		6.000 ± 0.0	
HDL-C (mg/dL)	57.75 ± 4.171	39.25 ± 0.7500	**	37.25 ± 0.6292	**
GLU (mg/dL)	216.3 ± 22.98	180.5 ± 8.930		197.8 ± 11.92	

Values are mean ± s.e.m. Blood was obtained from chow-fed 8-wk-old male mice after 4 h fasting.

*p < 0.05;

**p < 0.01;

***p < 0.001 compared with WT mice.
