# Supplementary material for: MicroRNA-33b knock-in mice for an intron of sterol regulatory element-binding factor 1 (Srebf1) exhibit reduced HDL-C in vivo
Source: Sci Rep. 2014 Jun 16;4:5312. doi: 10.1038/srep05312 (PMC4058878; doi:10.1038/srep05312)
Supplement: Supplementary Information [file srep05312-s1.pdf]

# Supplementary Information

# MicroRNA-33b knock-in mice for an intron of sterol regulatory element-binding factor 1 (*Srebf1*) exhibit reduced HDL-C *in vivo*

**Takahiro Horie, Tomohiro Nishino, Osamu Baba, Yasuhide Kuwabara, Tetsushi Nakao, Masataka Nishiga, Shunsuke Usami, Masayasu Izuhara, Fumiko Nakazeki, Yuya Ide, Satoshi Koyama, Naoya Sowa, Naoya Yahagi, Hitoshi Shimano, Tomoyuki Nakamura, Koji Hasegawa, Noriaki Kume, Masayuki Yokode, Toru Kita, Takeshi Kimura, and Koh Ono**

Supplementary Table S1

Serum lipid profiling of WT and miR-33b $KI^{+/+}$ mice by HPLC

| | Lipoprotein<br>Major (Fraction No.)<br>Diameter | Sub-class (Fraction No.) | WT (n=4) | $KI^{+/+}$ (n=5) |
|---|---|---|---|---|
| TC (mg/dl) | | | 72.71 ± 5.42 | 52.56 ± 3.91* |
| | CM (1-2) >80nm | | 0.73 ± 0.14 | 0.36 ± 0.06* |
| | VLDL (3-7) 30-80nm | | 3.93 ± 0.22 | 3.76 ± 0.41 |
| | LDL (8-13) 16-30nm | | 10.86 ± 0.60 | 9.85 ± 0.78 |
| | | large LDL (8) | 3.10 ± 0.09 | 3.39 ± 0.24 |
| | | medium LDL (9) | 2.85 ± 0.11 | 3.15 ± 0.21 |
| | | small LDL (10) | 1.77 ± 0.14 | 1.63 ± 0.18 |
| | | very small LDL (11-13) | 2.73 ± 0.60 | 1.68 ± 0.26 |
| | HDL (14-20) 8-16nm | | 57.20 ± 4.92 | 38.59 ± 3.23* |
| | | very large HDL (14-15) | 2.30 ± 0.50 | 0.68 ± 0.14* |
| | | large HDL (16) | 18.49 ± 2.44 | 8.89 ± 1.38** |
| | | medium HDL (17) | 24.90 ± 1.46 | 18.97 ± 1.29* |
| | | small HDL (18) | 8.45 ± 0.43 | 7.74 ± 0.47 |
| | | very small HDL (19-20) | 3.05 ± 0.25 | 2.32 ± 0.25 |
| TG (mg/dl) | | | 22.04 ± 2.48 | 19.27 ± 2.66 |

Values are mean ± SEM. Blood was obtained from chow-fed 8 weeks old male mice, after 4 h fasting.

The serum was analyzed by HPLC, as described in material and methods. *p<0.05, **p<0.01 compared with WT mice.

TC, total cholesterol; TG, triglyceride; CM, chylomicrons; VLDL, very low-density lipoprotein; LDL, low-density lipoprotein; HDL, high-density lipoprotein.

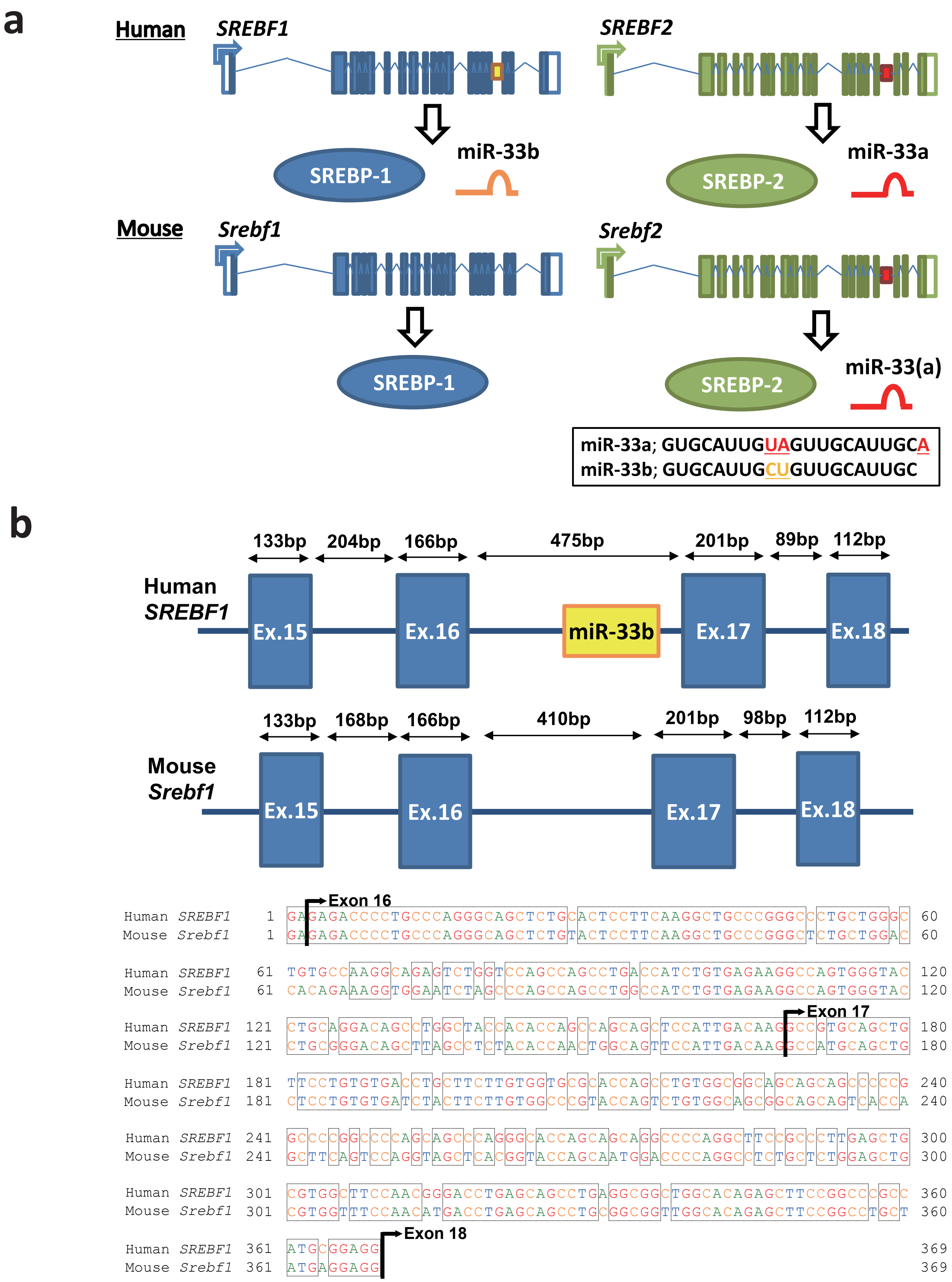


**Supplementary Figure S1. Scheme of *SREBFs* and miR-33s and homologies in exons 16 and 17 of *SREBF1* between human and mouse.** (a) miR-33b is lacking in intron of mouse *Srebf1*. Mature sequences of miR-33a and miR-33b are indicated in the inset. (b) There are high homologies in exons 16 and 17 between human *SREBF1* and mouse *Srebf1* (82.6% of nucleotides and 79.7% of amino acids).

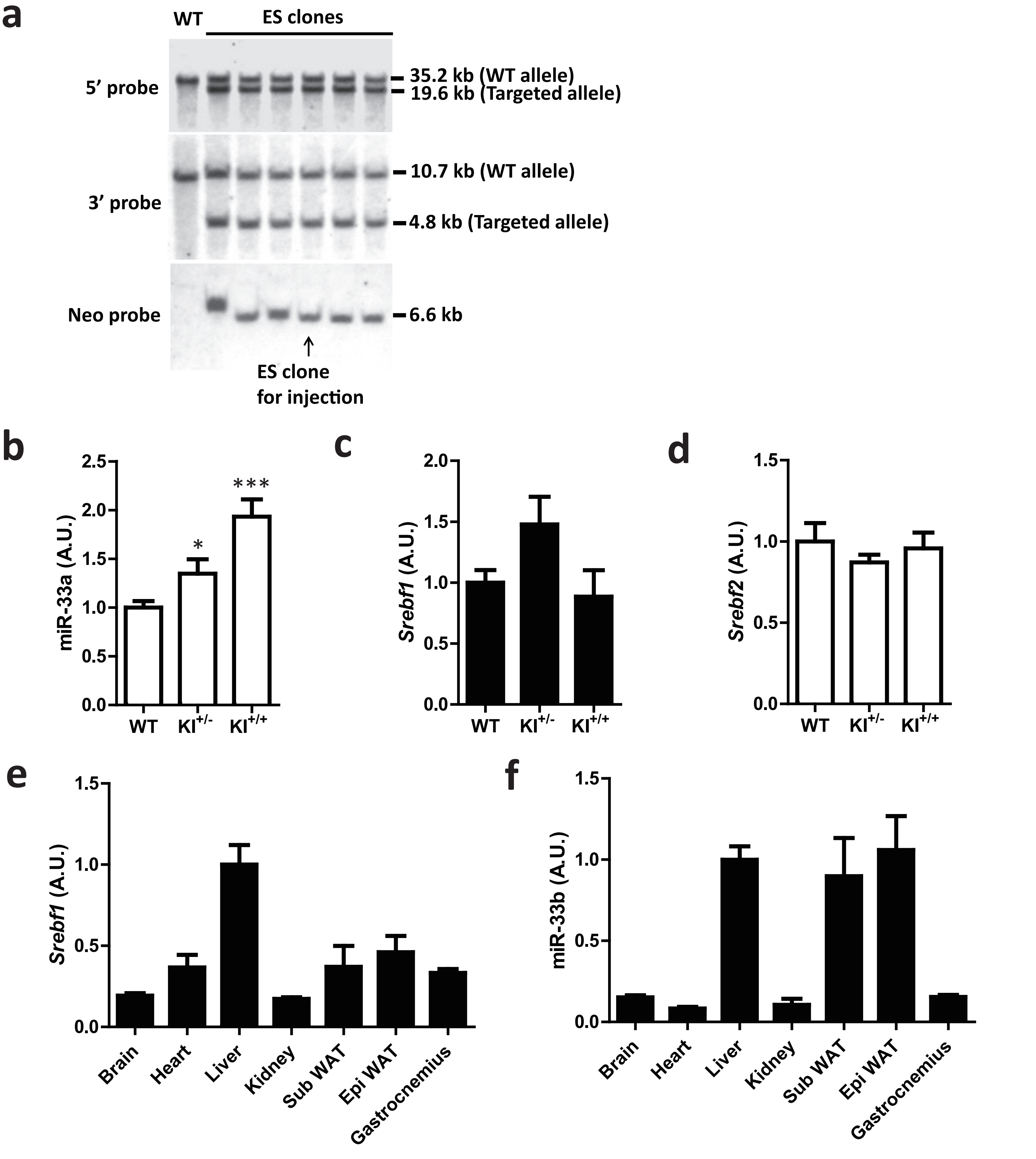


**Supplementary Figure S2. Generation of miR-33b knock-in mice.** (a) Southern blotting analysis of genomic DNA from ES cells that were successfully targeted by a KI vector. Representative images are shown. (b-d) Quantitative real-time PCR analysis of miR-33a (b), *Srebf1* (c) and *Srebf2* (d) in the liver of 8-wk-old WT, $KI^{+/-}$ and $KI^{+/+}$ male mice (n=6). Values are mean ± s.e.m. *p < 0.05 ***p < 0.001 by one-way analysis of variance. (e, f) Quantitative real-time PCR analysis of *Srebf1* (e) and miR-33b (f) in 8-wk-old $KI^{+/+}$ male mice (n=3). Values are mean ± s.e.m. Sub WAT indicates subcutaneous white adipose tissue. Epi WAT indicates epididymal white adipose tissue.

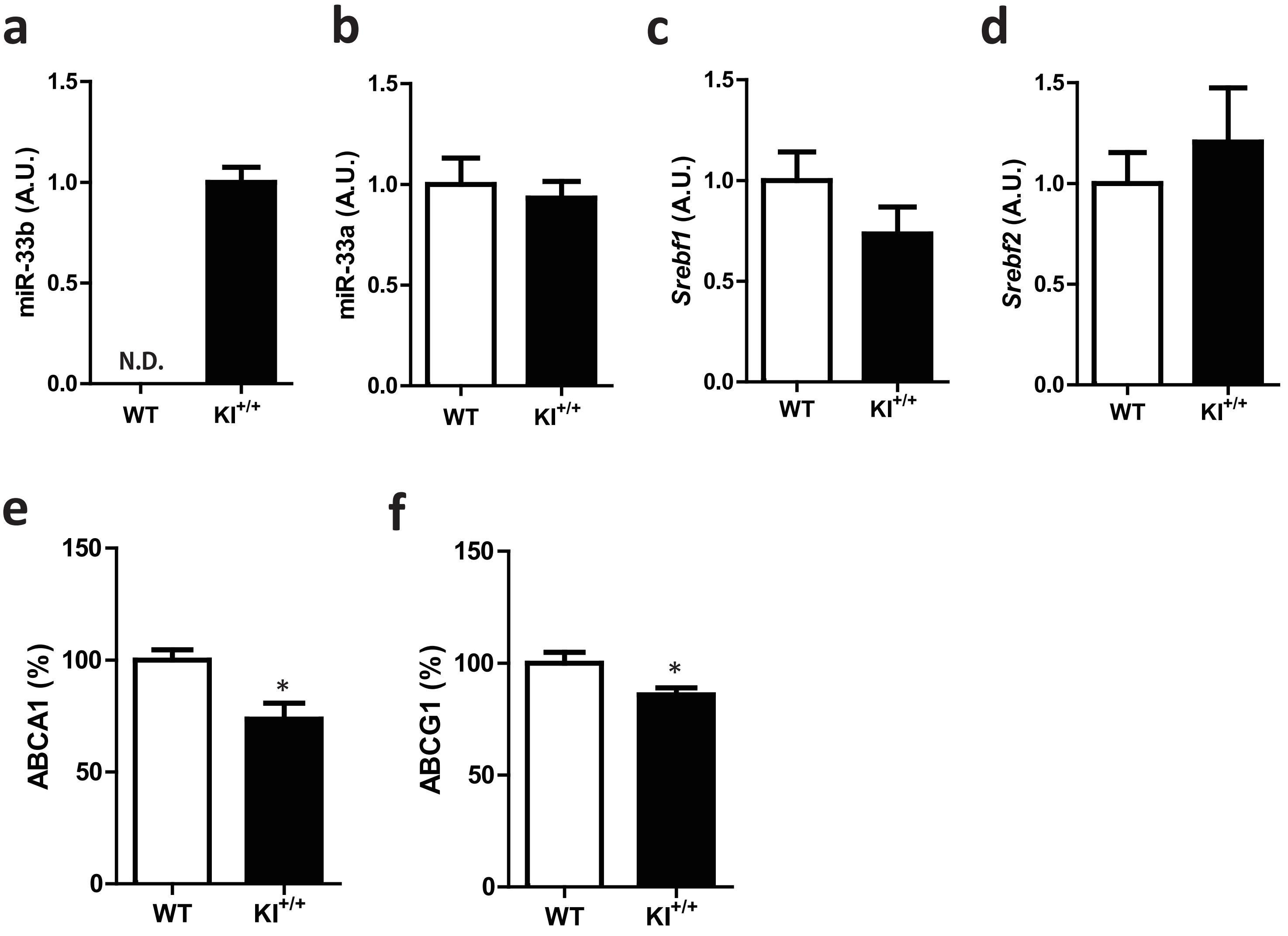


**Supplementary Figure S3. The expression levels of miR-33s and *Srebfs* in KI macrophages.** (a-d) Quantitative real-time PCR analysis of miR-33b (a), miR-33a (b), *Srebf1* (c) and *Srebf2* (d) in peritoneal macrophages from 8-wk-old WT and $KI^{+/+}$ male mice (n=3). Values are mean ± s.e.m. N.D., not determined. (e, f) Densitomeric analysis of ABCA1 (e) and ABCG1 (f) in peritoneal macrophages from 8-wk-old WT and $KI^{+/+}$ male mice. (n = 4). Values are mean ± s.e.m. *$p < 0.05$ by Student's t-test.

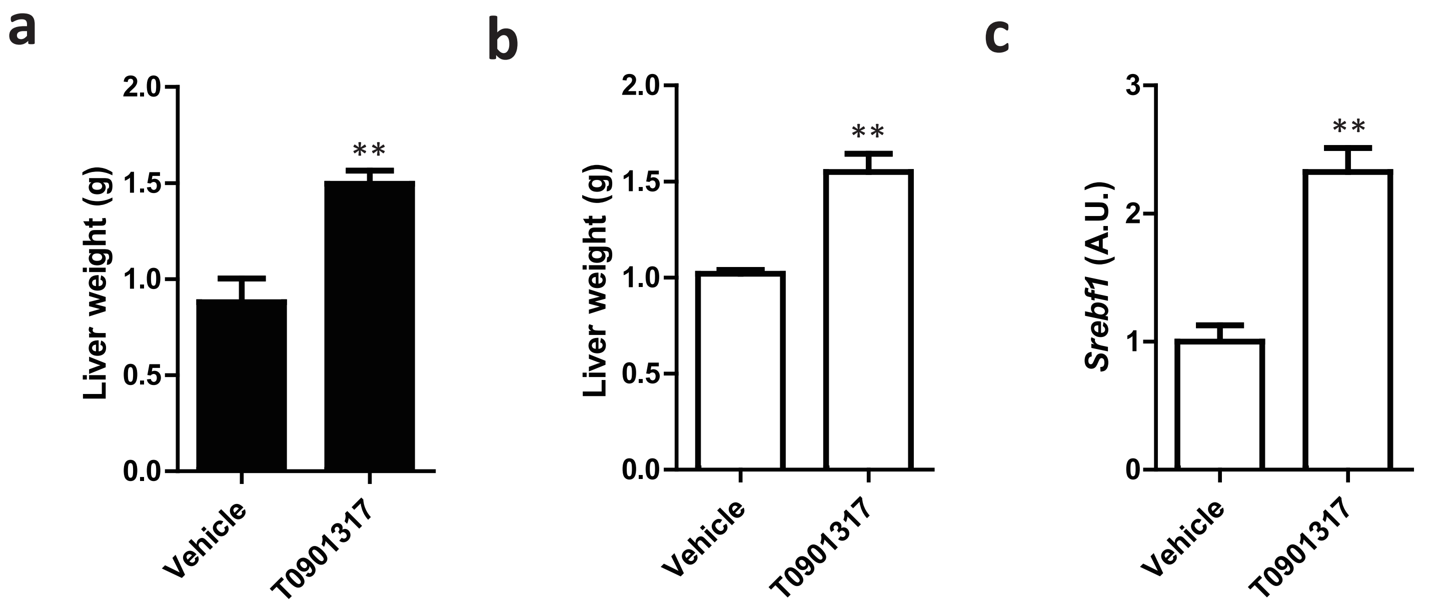


**Supplementary Figure S4. Liver weight and *Srebf1* expression of T0901317-treated mice.** (a) Liver weight of vehicle or T0901317-treated 8-wk-old $KI^{+/+}$ male mice. Values are the means ± s.e.m. (n = 6, **p<0.01 by Student's t-test). (b) Liver weight of vehicle or T0901317-treated 8-wk-old WT male mice (n=4, **p<0.01 by Student's t-test). (c) Quantitative real-time PCR analysis of *Srebf1* in the liver of vehicle or T0901317-treated 8-wk-old WT male mice (n=4, **p< 0.01 by Student's t-test).

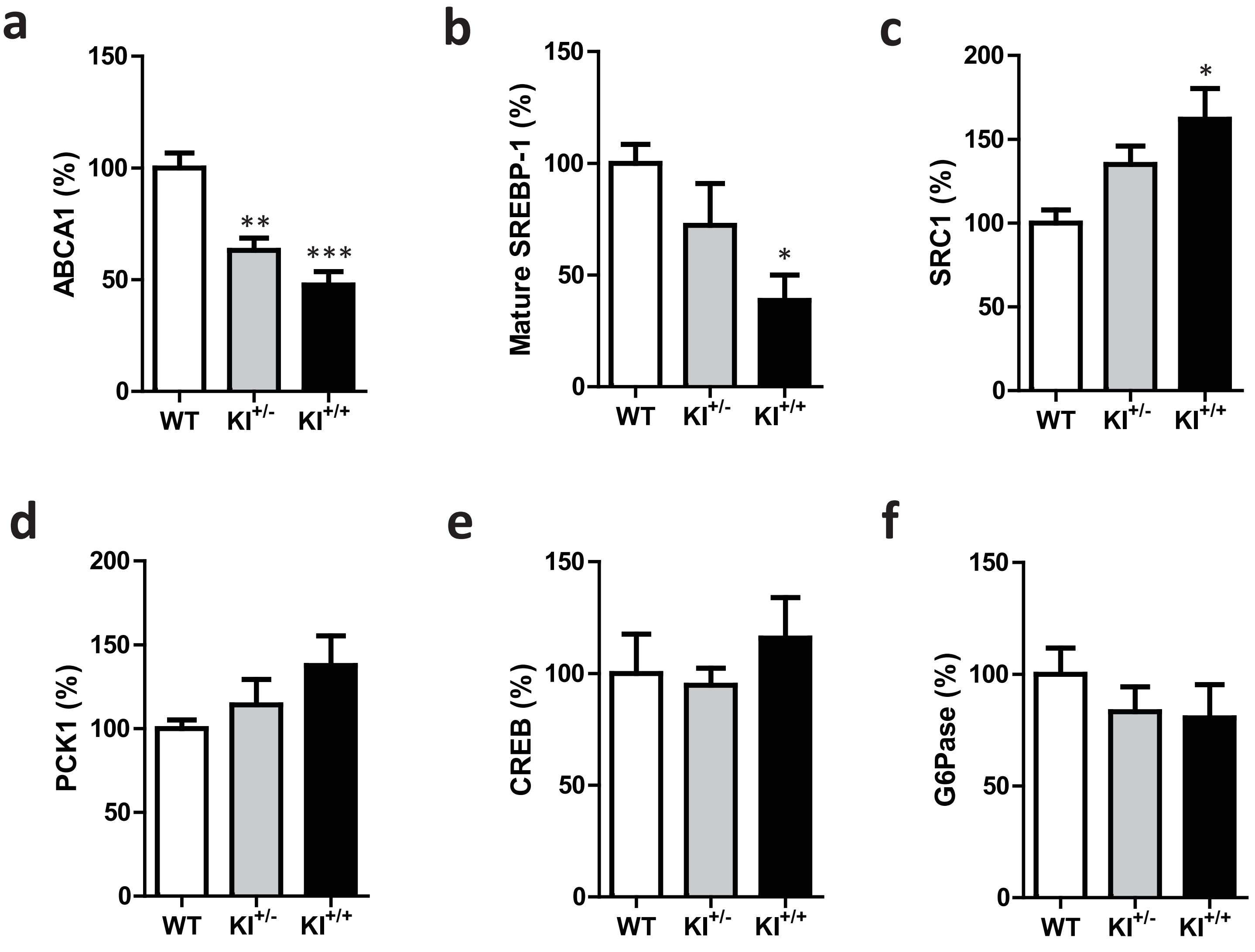


**Supplementary Figure S5. The expression levels of cholesterol and glucose metabolic genes.** The protein expression levels of ABCA1 (a), SREBP-1 (b), SRC1 (c), PCK1 (d), CREB (e) and G6Pase (f) in the liver of 8-wk-old WT, $KI^{+/-}$ and $KI^{+/+}$ male mice by densitometry. The expression levels were normalized with β-actin or TF2B expressions. The expression levels of WT mice were set at 100%. Values are mean ± s.e.m. *$p < 0.05$, **$p<0.01$ ***$p < 0.001$ compared with WT by one-way analysis of variance (a-d; n=6, e and f; n=3).

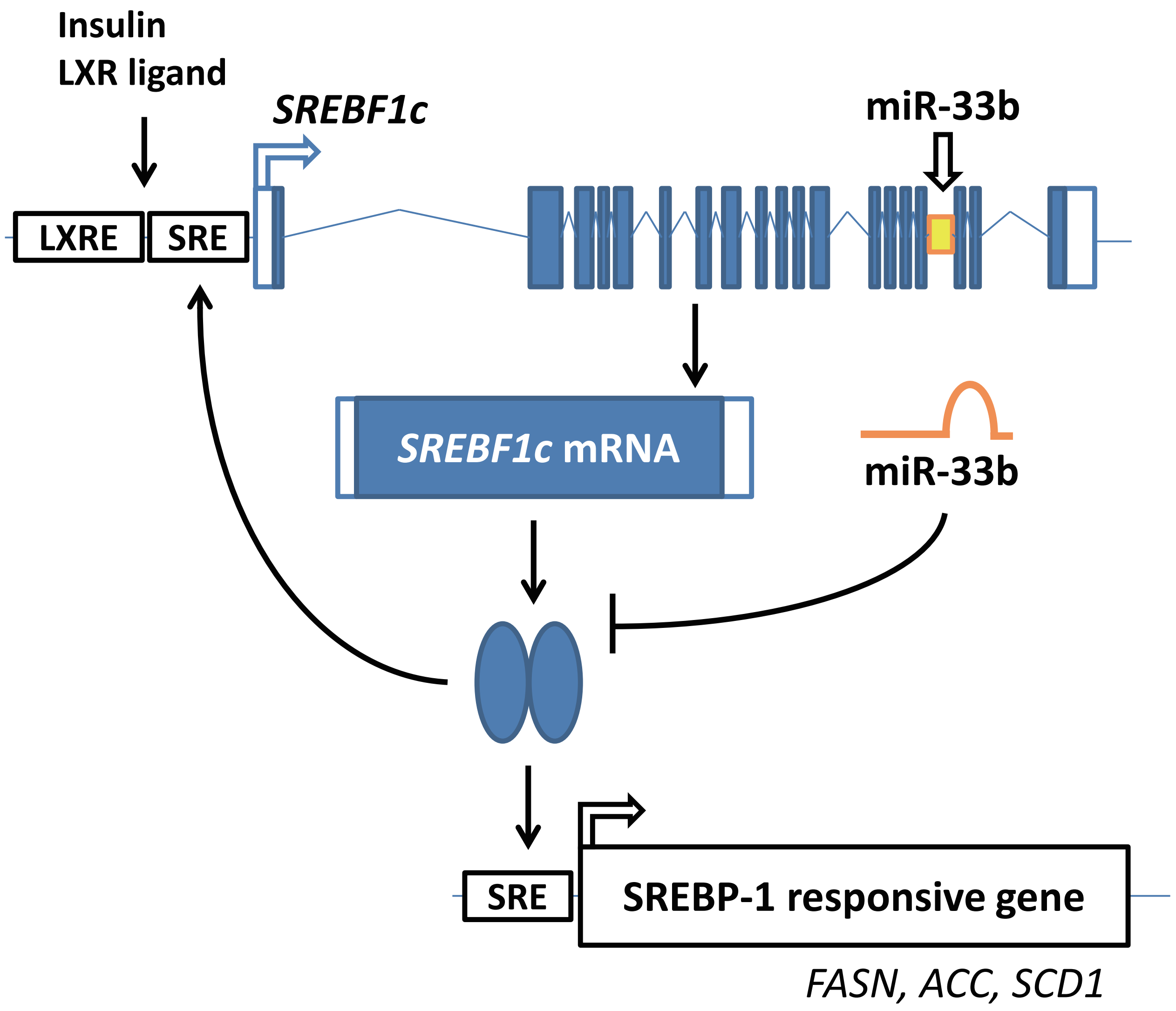


**Supplementary Figure S6. Proposed feedback mechanism to regulate SREBP-1 level by intronic miR-33b.**

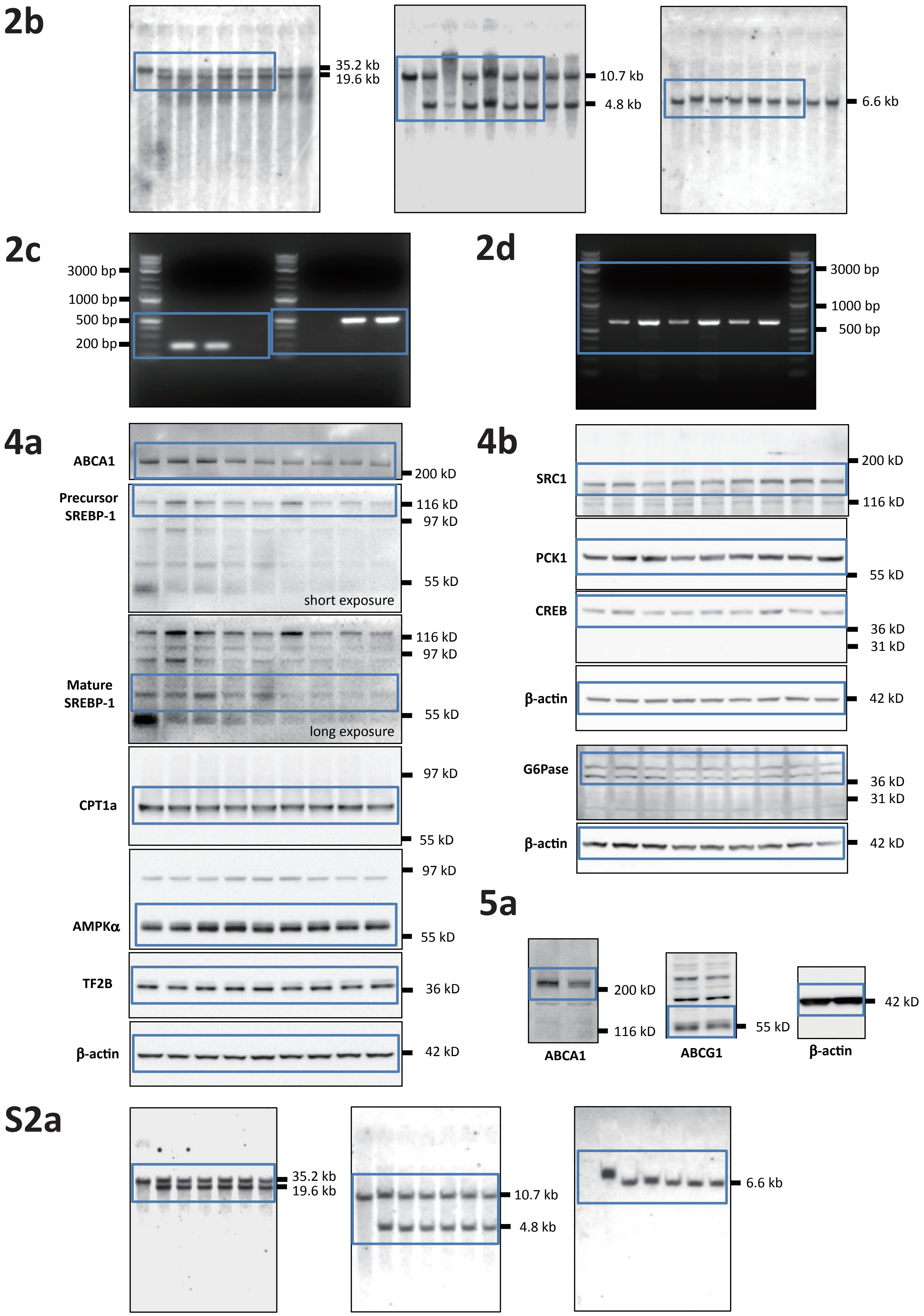


**Supplementary Figure S7. Scans of the original blots in figure 2b, 2c, 2d, 4a, 4b, 5a and S2a.**
